# Frequency of subclavian artery stenosis in patients with mammarian artery coronary bypass and suspected coronary artery disease progression

**DOI:** 10.1007/s00392-022-02113-z

**Published:** 2022-10-14

**Authors:** Arne M. Müller, Justus Bertram, Christian Bradaric, Tobias Koppara, Salvatore Cassese, Erion Xhepa, Britta Heilmeier, Ilka Ott, Adnan Kastrati, Karl-Ludwig Laugwitz, Tareq Ibrahim, Ralf J. Dirschinger

**Affiliations:** 1 Klinik Und Poliklinik Für Innere Medizin I., Klinikum Rechts Der Isar, Technische Universität München, Ismaninger Str. 22, 81675 Munich, Germany; 2 Deutsches Herzzentrum München, Abteilung Für Herz- Und Kreislauferkrankungen, Technische Universität München, Lazarettstr. 36, 80636 Munich, Germany; 3 Gefäßpraxis im Tal, Tal 13, 80331 Munich, Germany; 4 Helios Klinikum Pforzheim, Abteilung für Kardiologie, Angiologie und Intensivmedizin, Kanzlerstr. 2‐6, 75175 Pforzheim, Germany; 5 DZHK (German Centre for Cardiovascular Research), Partner Site Munich Heart Alliance, Biedersteiner Str. 29, 80331 Munich, Germany

**Keywords:** Atherosclerosis, Subclavian artery, Coronary bypass, Angioplasty, Endovascular procedures

## Abstract

We retrospectively analyzed patient records of all patients with a history of internal mammarian artery (IMA) coronary bypass undergoing coronary angiography at two cardiovascular centers between January 1st 1999 and December 31st 2019. A total of 11,929 coronary angiographies with or without percutaneous coronary intervention were carried out in 3921 patients. Our analysis revealed 82 (2%) patients with documented subclavian artery stenosis. Of these, 8 (10%) patients were classified as having mild, 18 (22%) moderate, and 56 (68%) severe subclavian artery stenosis. In 7 (9%) patients with subclavian artery stenosis, angiography revealed occlusion of the IMA graft. 26 (32%) patients with severe subclavian artery stenosis underwent endovascular or surgical revasculararization of the subclavian artery. In this retrospective multicenter study, subclavian artery stenosis was a relevant finding in patients with an internal mammarian artery coronary bypass graft undergoing coronary angiography. The development of dedicated algorithms for screening and ischemia evaluation in affected individuals may improve treatment of this potentially underdiagnosed and undertreated condition.

**Supplementary Information:**

The online version contains supplementary material available at 10.1007/s00392-022-02113-z.

## Introduction

A significant portion of patients with coronary artery disease (CAD) also suffers from atherosclerotic disease of peripheral or cerebral arteries [1]. While the presence of polyvascular disease generally indicates higher morbidity, concomitant peripheral artery or cerebrovascular disease does not directly affect myocardial perfusion. This is different in patients with CAD and upper extremity arterial disease who have a history of coronary artery bypass grafting utilizing the internal mammary artery (IMA). In these patients, a stenosis of the ipsilateral subclavian artery can directly cause myocardial ischemia. Findings range from reduced flow in the IMA graft over stress induced or permanent flow reversal in the graft (termed subclavian coronary steal syndrome) [2] to bypass graft occlusion following chronic flow stagnation. The consequence is myocardial ischemia during physical exercise or during ipsilateral manual work, unstable angina, myocardial infarction and silent ischemia with congestive heart failure [3, 4].

Subclavian stenosis per se can lead to vertebral artery steal with neurologic symptoms. Yet, in the majority of cases subclavian stenosis is an asymptomatic finding and appears to be generally benign under medical therapy in the absence of an IMA graft [5–7]. For this reason, the condition is likely underdiagnosed and has been systematically addressed in treatment guidelines only in the recent past. Conservative treatment is the primary choice for many patients in clinical practice. However, this may be a poor treatment choice when coronary perfusion is affected. Nevertheless, recommendations for the diagnosis, evaluation, and treatment of subclavian stenosis in patients with IMA graft and symptoms of CAD progression are scarce and not standardized.

Several studies have estimated the prevalence of this condition in different pre- or postoperative populations, ranging from 2 to 7%, most of them with small patient numbers [8–13]. To assess the frequency of this potentially underdiagnosed and undertreated condition in an all-comers population, we retrospectively analyzed the medical records of almost 4000 patients with IMA coronary bypass grafts presenting for coronary angiography at two major cardiovascular centers in Munich, Germany, between 1999 und 2019 for evidence of subclavian artery stenosis.

## Methods

### Patients

All patients with a history of internal mammarian artery (IMA) coronary bypass undergoing coronary angiography at two university cardiac centers between January 1st 1999 and December 31st 2019 were identified using the cath labs’ database system and retrospectively analyzed. Indications for coronary angiography were myocardial infarction (STEMI or NSTEMI), unstable and stable angina pectoris, atypical symptoms and other indications [14]. All participants provided written informed consent for the clinical procedure. In accordance with the local ethics committee, this purely retrospective analysis did not require additional informed consent.

### Database matching

In addition to the cath laboratory database, patient data from the centers’ electonic data management system and electronic files were retrieved using the search terms subclavian artery, LIMA, RIMA and mammarian for the given timespan (s. above). Moreover, all patients undergoing subclavian artery percutaneous transluminal angioplasty (PTA) were analyzed for the presence of coronary artery bypass grafts and symptoms. All search results were compared and matching patients with subclavian artery stenosis were identified using patient name and date of birth (Supplemental Fig. 1).

### Subclavian stenosis diagnosis and graduation

Subclavian stenoses in this analysis were graduated as mild, moderate, or severe. The semiquantitave graduation reflects general clinical practice and was taken from the medical files or cath lab reports as documented by the treating clinicians at the time of patient care. Coronary angiography in patients with IMA-bypass usually includes selective angiography of the IMA with catheter placement over the subclavian artery, generally with continous invasive blood pressure recording at the catheter tip during catheter placement and pull-back, which allows the detection of pressure gradients. Feasibility of subclavian passage by catheter, absence of a pressure gradient between aortic arch and subclavian artery, and/or presence of a strong competitive flow in the bypassed coronary vessel are generally considered to rule out significant subclavian stenosis. Therefore, subclavian angiography or aortic arch angiography is generally only performed during coronary angiography at the interventionalists discretion when subclavian stenosis is suspected and for the majority of patients no direct subclavian angiography was reported. Documented diagnoses and graduation in the study population were reportedly based on the percentage of angiographic stenoses (including MRA and CTA), duplex ultrasound peak flow velocities, invasive and non-invasive pressure gradients and/or flow in distal arteries/flow reversal in distal side branches. Reported imaging modalities are outlined in the results section.

### Statistical analysis

The predefined primary endpoint was the presence of subclavian artery stenosis as documented in the medical record, usually determined by angiography, duplex ultrasound (DUS), MR or CT angiography, or a combination of these methods. Stenoses were classified as mild, moderate, and severe according to the medical records. Characteristics of the patients, lesions and coronary angiographies with or without percutaneous coronary intervention (PCI) were summarized using descriptive statistics: mean and standard deviation for quantitative data and frequency (%) for qualitative data. The chi-squared test was used to compare distributions of categorical data, including the primary endpoint, between independent groups. The *t*-test for independent samples was used for group comparisons regarding continuous data. Data were analyzed using Microsoft Excel for Mac 16.53 and IBM SPSS Statistics 26 and presented following general recommendations [15].

## Results

### Overall patient and procedural characteristics

A total of 11,929 coronary angiographies in 3,921 patients with IMA-bypass grafts were identified. Patient characteristics are shown in Table 1. Procedural Characteristics of coronary angiographies with or without PCI and Clinical Presentation are summarized in Table 2. Average patient age was 81 ± 10.3 years and 3215 (82%) patients were male. The most common cardiovascular risk factors were hypertension and hypercholesterolemia, which were present in 3467 (88%) and 2877 (73%) patients, respectively. 1283 (33%) patients suffered from diabetes, 1473 (38%) were current or former smokers, and 1177 (30%) had a family history of cardiovascular disease. A previous myocardial infarction or a history of PCI were found in 1529 (39%) and 1424 (36%) patients, respectively. About 2324 (59%) patients received diagnostic coronary angiography and 1597 (40%) underwent PCI at least once (Supplemental Fig. 2). In 2296 (19%) angiographies, the indication was an acute coronary syndrome and in 4681 (39%), a typical stable angina pectoris. Our analysis revealed 82 (2%) patients with a stenosis of the subclavian artery. Of the 82 patients with subclavian artery stenosis, 62 (76%) received invasive subclavian angiography, 17 (21%) underwent MRA (magnetic resonance angiography), and 10 (12%) underwent CTA (computed tomography angiography). In the 20 patients without documented invasive angiography, diagnosis was established in 7 patients by MRA, in 6 patients by CTA (computed tomography angiography), and in 7 patients by duplex sonography only. Thus, 75 patients (91%) received at least one angiographic imaging modality to confirm diagnosis in addition to duplex ultrasound and non-invasive blood pressure measurements.

**Table 1:** Patient characteristics (*n*/*N* (%) unless otherwise stated)

	All patients (*n* = 3921)	Patients with subclavian stenosis and ipsilateral IMA-bypass (*n* = 82)	P*
Age (years) Mean ± SD	81.34 ± 10.3	82.41 ± 10.5	0.113
Sex			
Male	3215/3921 (82)	53/82 (65)	< 0.0001
Female	706/3921 (18)	29/82 (35)	
Risk factors			
Hypertension	3467/3921 (88)	66/82 (80)	0.023
Hypercholesterolemia	2877/3921 (73)	55/82 (67)	0.192
Diabetes	1283/3921 (33)	23/82 (28)	0.362
Current or former smoker	1473/3921 (38)	32/82 (39)	0.783
Family history	1177/3921 (30)	21/82 (26)	0.379
Cardiac history			
LIMA bypass	3297/3921 (84)	74/82 (90)	0.123
RIMA bypass	63/3921 (2)	5/82 (6)	0.001
LIMA and RIMA bypass	561/3921 (14)	3/82 (4)	0.005
Previous MI	1529/3921 (39)	36/82 (44)	0.357
Previous PCI	1424/3921 (36)	33/82 (40)	0.455
Pat. with diagnostic angiography	2324/3921 (59)	43/82 (52)	0.203
Pat. with PCI	1597/3921 (41)	39/82 (48)	0.203

*IMA* internal mammarian artery, *LIMA* left IMA, *RIMA* right IMA, *MI* myocardial infarction, *PCI* percutaneous coronary intervention

*p values for comparison of patients with subclavian stenosis and patients without stenosis

**Table 2:** Procedural characteristics and clinical presentation (*n*/*N* (%) unless otherwise stated)

	All coronary angiographies with or without PCI (*n* = 11,929)	Coronary angiographies with or without PCI in pts. with subclavian stenosis and ipsilateral IMA-bypass (*n* = 254)	P*
Diagnostic angiography	6949/11929 (58)	147/254 (58)	0.901
PCI	4980/11929 (42)	107/254 (42)	0.901
Clinical presentation (per procedure)			
ACS	2296/11929 (19)	52/254 (20)	0.617
STEMI	116/11929 (1)	4/254 (2)	0.323
NSTEMI	1095/11929 (9)	19/254 (7)	0.343
Unstable Angina	1085/11929 (9)	29/254 (11)	0.193
Stable Angina	4681/11929 (39)	110/254 (43)	0.18
Atypical Angina	1256/11929 (11)	21/254 (8)	0.235
Pathological stress imaging	450/11929 (4)	5/254 (2)	0.127
Pathological exercise ECG	1062/11929 (9)	23/254 (9)	0.931
Follow-up angiography after PCI	1164/11929 (10)	36/254 (14)	0.017
Other indication	1020/11929 (9)	7/254 (3)	< 0.001

*IMA* internal mammarian artery, *PCI* percutaneous coronary intervention, *ACS* acute coronary syndrome, *STEMI* ST-segment elevation myocardial infarction, *NSTEMI* Non-ST-segment elevation myocardial infarction

*p values for comparison of angiographies (w/wo PCI) in patients with subclavian stenosis and angiographies (w/wo PCI) in patients without stenosis

### Characteristics of patients with subclavian stenosis and IMA-bypass

In 82 patients with a history of IMA-bypass, a definite stenosis of the subclavian artery was diagnosed as documented in the medical record. 53 (65%) patients were male, 36 (44%) had a history of myocardial infarction, and 33 (40%) had a history of PCI. Of these 82 patients, 4 presented with ST-segment elevation myocardial infarction, 10 with Non-ST-segment elevation myocardial infarction, 17 with unstable angina, 48 with stable angina, and 3 with atypical symptoms at least once as the most severe clinical reason for presentation (in cases of multiple visits). Compared to the overall population, the proportion of female patients was significantly larger in the subset with subclavian stenosis (29/82 vs. 706/3921; 35.4% vs. 18.1%; *p* < 0.0001). Hypertension was seen less frequently in patients with subclavian stenosis (66/82 vs. 3467/3921; 80.5% vs. 88.4%; *p* = 0.023). No relevant differences in age, other risk factors, or cardiac history were observed.

### Classification, symptoms, and treatment of subclavian artery stenosis

**Fig. 1:**
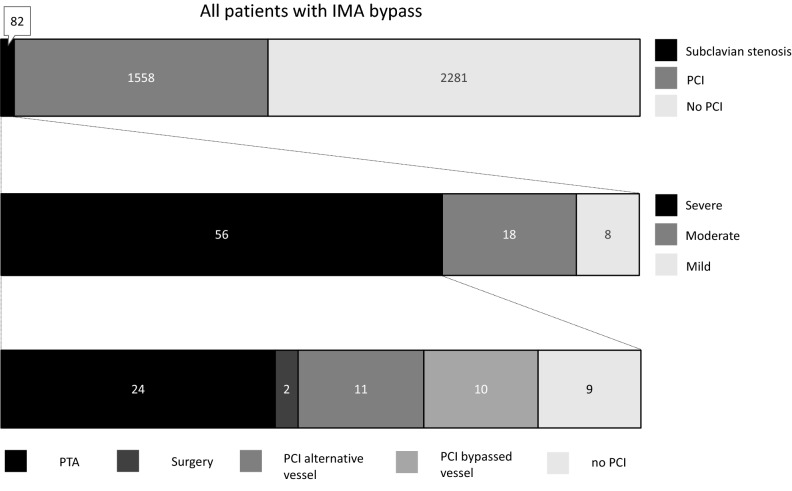
Prevalence, classification and treatment of subclavian stenosis in patients with IMA-bypass and Indication for coronary angiography

Subclavian artery stenosis lesion classification and subsequent treatment are summarized in Table 3 and Fig. 1. 26 of 82 (32%) lesions were classified as mild or moderate stenoses, none of which received invasive treatment of the lesion. 56 (68%) lesions were classified as severe subclavian stenoses.

**Table 3:** Classification of subclavian stenosis and subsequent treatment in patients with subclavian stenosis and ipsilateral IMA-bypass graft. (*n/N (%)*)

Grade of stenosis	
Mild	8/82 (10)
Moderate	18/82 (22)
Severe, invasive treatment	26/82 (32)
PTA of subclavian artery	24/82 (29)
Surgery on subclavian artery	2/82 (2)
Severe, conservative treatment	30/82 (37)
PCI in alternative vessel	11/82 (13)
PCI in native bypassed vessel*	10/82 (12)
No PCI	9/82 (11)
LIMA/RIMA graft occluded	7/82 (9)

*IMA* internal mammarian artery, *LIMA* left internal mammarian artery, *RIMA* right internal mammarian artery, *PCI* percutaneous coronary intervention, *PTA* percutaneous transluminal angioplasty

*Current or previous

In patients diagnosed with severe subclavian stenosis, 40 presented with only cardiac symptoms (71% of severe subclavian stenosis; chest pain or dyspnea), two patients with only neurological symptoms (4%, vertigo or syncope), three patients with only brachial claudication (5%), one patient with both neurologic symptoms and brachial claudication (2%), five patients with both cardiac and neurologic symptoms or brachial claudication (9%), and in five patients, no typical symptoms were documented (9%). Several patients had concomitant conditions that may also have been responsible for the symptoms, i.e., coronary artery stenosis or valvular heart disease.

**Fig. 2:**
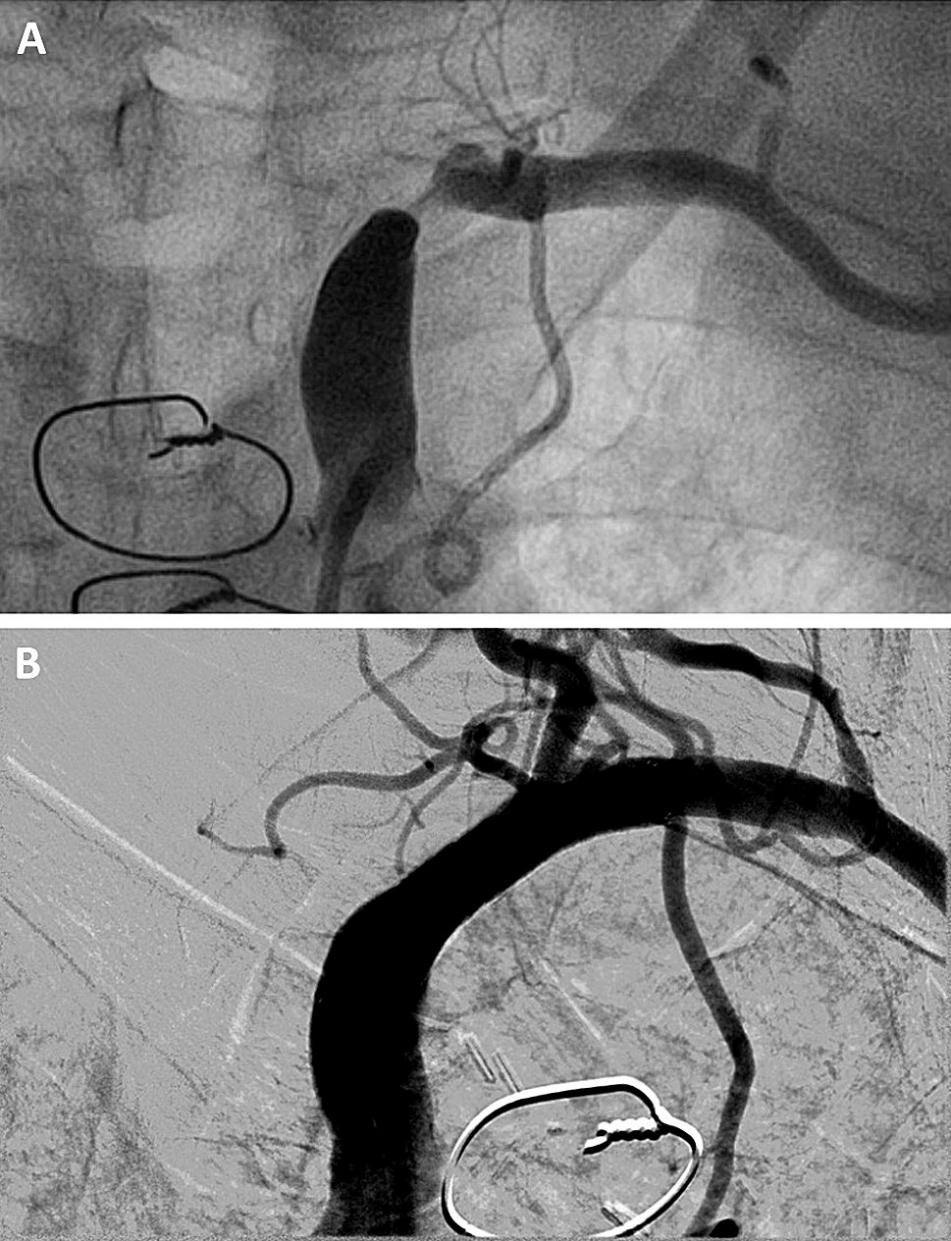
**A** Angiography revealing severe stenosis of the left subclavian artery in a 77 year old male patient with IMA-bypass to the LAD. **B** Digital subtraction angiography after successful revascularization using PTA and stenting

26 of the patients with severe subclavian stenosis (46% of severe subclavian stenoses) received invasive treatment, comprising of PTA and stenting of the subclavian artery in 24 and vascular surgery on the subclavian artery in two cases. Representative images of a patient treated with PTA are shown in Fig. 2. In the 24 patients treated by PTA at the two institutions, no major complications were documented. The remaining 30 patients (54% of patients with severe stenosis) received no invasive treatment of the subclavian lesion. Within this group, 11 patients underwent PCI of an alternative vessel as primary target lesion, 10 patients were treated with a PCI in the native coronary vessel receiving the IMA graft and in 9 patients no PCI was carried out. In the patients treated with PCI, three cases suffering major complications were documented: One patient experienced transitory ischemic attack following PCI. One patient suffered access site bleeding requiring transfusion and one patient presenting with STEMI and cardiogenic shock died. In patients with severe subclavian stenosis, the average timespan from the IMA-bypass operation to the diagnosis of the subclavian stenosis was 10.40 ± 6.18 SD (years).

Lastly, in 7 of patients with severe subclavian stenosis (13%), all in the group receiving no subclavian intervention, the LIMA or RIMA graft was occluded.

## Discussion

In the current study, we retrospectively analyzed a large, multicenter patient cohort with IMA coronary bypass presenting for coronary angiography and suspected CAD progression over a period of more than 20 years for the presence of subclavian stenosis. With data from almost 4000 individuals, this study presents the largest population addressing this question to this date.

### Prevalence of subclavian stenosis

Subclavian stenoses ipsilateral to the IMA grafts were present in about 2% of patients, two-thirds of which were classified as severe. The prevalence of subclavian stenosis in the general population is reported at about 2–4% and may be as high as 7–18% in patients with PAD [8, 16, 17]. In several studies screening for subclavian stenoses in patients with CAD referred for potential CABG surgery, the prevalence was 2.5–6.8% [8, 10, 12, 13] with 2.5% in the largest population (1,498 patients) [12].

There are different explanations for the slightly lower frequency in our study: first, our population differs significantly when compared to the published pre-CABG populations in several regards. On the one hand, patients with significant subclavian stenosis may have been treated or excluded from IMA-bypass grafting prior to surgery in our study. State-of-the-art pre-CABG diagnostics provided, most of the subclavian stenoses identified in our study should be de novo stenoses occuring after bypass surgery. However, the subclavian stenoses in our study may in part reflect the prevalence of lesions that were not detected before CABG surgery and in part the new incidence of stenoses that developed after CABG surgery.

On the other hand, average patient age in our population was 80 years and therefore older than published pre-CABG populations, which may positively or negatively affect prevalence and incidence of subclavian stenoses. While an older population would generally be considerd to have an increased burden of polyvascular disease, the condition studied may also have a negative prognostic impact over time.

Secondly, our study was purely retrospective and no prospective screening procedure was performed. Thus, it is possible that subclavian stenoses were missed during the clinical workup of patients. Particularly mild and moderate stenoses may not have been identified by non-invasive blood pressure measurement, invasive blood pressure measurement during coronary angiography, and particulary when performing only selective IMA angiography.

Interestingly, we saw an increase in the number of patients diagnosed with subclavian stenosis over time (Supplemental Fig. 3). This documented rise is most likely caused by an increased awareness of this relevant medical condition in everyday clinical practice, improved screening algorithms including the implementation of routine bilateral blood pressure measurement in all patients, and improved availability of high quality duplex ultrasound. Considering this change in diagnostic prevalence over time, we cannot rule out that significant stenoses may have been missed. In patients with subclavian stenoses, female gender was more frequent compared to the overall population (35% vs. 18%). The reasons for this difference may be related to vessel size, sex differences in vascular biology, or to chance in our population. Further analyses are required for a improved understanding of sex differences in the presentation and pathophysiology of vascular disease.

### Treatment strategies

Endovascular treatment is feasible for many patients with subclavian stenosis, and endovascular and surgical treatments generally achieve good results at low complication rates [5, 12, 18]. In the current study, only half of the patients with severe subclavian stenosis received endovascular or operative revascularization of the subclavian artery. Within the other half, two-thirds underwent PCI either in the native coronary vessel receiving the bypass, thereby compensating for the insufficient IMA graft function, or PCI in a different coronary vessel considered the target lesion at the time of diagnosis. The remaining third (11% of patients with ipsilateral subclavian artery stenosis) did not receive any revascularization procedure. Documented reasons for a complete conservative treatment decision included negative ischemia testing, insufficient or occluded IMA graft, limited putative benefit, and failure to present for scheduled ischemia testing or revascularization. In some cases, no reason was documented. Considering age and morbidity of the patient population, conservative treatment numbers appear acceptable. Nevertheless, the presence of occluded IMA grafts in 9% of patients with subclavian stenosis raises the question, if some of these IMA graft occlusions occurred as a consequence of the subclavian stenosis and could have been prevented by earlier diagnosis and treatment. 73% of patients with severe stenosis who received subclavian revascularisation reported symptom relief. This rate was numerically lower in the other groups, suggesting benefit from subclavian revascularistation, although numbers were too low to draw valid conclusions.

### Clinical significance and screening

Subclavian artery stenosis in patients with IMA grafts may present in various clinical forms, as sudden death, myocardial infarction, stable angina, or progressive LV dysfunction [3, 4]. Therefore, subclavian stenosis should always be considered in patients with IMA graft and cardiac symptoms. We believe that subclavian stenosis in patients with ipsilateral coronary artery IMA-bypass graft should never be considered a benign condition and requires systematic diagnostic testing and treatment in many cases. Depending on the supply area of the IMA graft, its prognostic significance may be comparable to a significant stenosis of the left main coronary artery in individuals without CABG.

Even so, due to the frequent lack of symptoms and the benign course of the condition in asymptomatic patients without coronary IMA graft, there is still limited awareness and likely a significant underdiagnosis of the condition, despite the simplicity of its diagnosis.

Diagnosis of subclavian stenosis is easily established by non-invasive testing. Bilateral blood pressure measurement and additional color duplex ultrasonography in case of ≥ 15 mmHg inter-arm blood pressure difference are simple and cost-effective screening tools with acceptable sensitivity for the identification of severe subclavian stenoses [19].

Finally, in patients with an IMA graft and known subclavian stenosis, guidelines recommend ischemia testing [6]. Yet, in our experience, treadmill exercise or pharmacologic testing may not detect clinically relevant subclavian steal phenomena induced by brachial exercise. Hand grip exercise testing can be used to detect clinically relevant ischemia [2, 20]. Development of standardized testing procedures for all patients with IMA grafts undergoing ischemia testing need to be established.

### Limitations

The current multicenter study has all important limitations related to its retrospective design. Subclavian stenoses were graduated semiquantitatively by the treating clinicians at the time of diagnosis based on several parameters. A large number of patients received more than one coronary angiography. The high average age and the population may not ideally represent other populations. Most importantly, lacking a prospective screening approach, we cannot rule out that subclavian stenoses were missed during the clinical workup of patients.

## Conclusions

In this large retrospective multicenter analysis post CABG surgery, subclavian artery stenosis proximal to an IMA graft was a relevant finding in patients undergoing coronary angiography. The use of dedicated algorithms for screening and ischemia evaluation in affected individuals may improve the treatment of this potentially underdiagnosed condition.

## Supplementary Information

Below is the link to the electronic supplementary material.

Supplementary file1 (DOCX 137 KB)
