# Supplementary material for: Frequency of subclavian artery stenosis in patients with mammarian artery coronary bypass and suspected coronary artery disease progression
Source: Clin Res Cardiol. 2022 Oct 14;112(9):1204–11. doi: 10.1007/s00392-022-02113-z (PMC10449982; doi:10.1007/s00392-022-02113-z)
Supplement: Supplementary file 1 — Supplementary file1 (DOCX 137 KB) [file 392_2022_2113_MOESM1_ESM.docx]

**Supplementary Appendix**

**Supplemental Figure 1.** Patient identification

**Supplemental Figure 2.** Study flowchart

**Supplemental Figure 3.** Frequency of diagnosis of subclavian stenosis.
